# Plasmonic lens focused longitudinal field excitation for tip-enhanced Raman spectroscopy

**DOI:** 10.1186/s11671-015-0897-0

**Published:** 2015-04-18

**Authors:** Mingqian Zhang, Jia Wang

**Affiliations:** Qian Xuesen Laboratory of Space Technology, China Academy of Space Technology, Youyi Road No. 104, Haidian, Beijing, 100094 China; State Key Laboratory of Precision Measurement Technology and Instruments, Department of Precision Instruments, Tsinghua University, 30 Shuang Qing Lu, Haidian, Beijing, 100084 China

**Keywords:** Tip-enhancement, Plasmonic lens, Longitudinal electric field, Surface plasmon polaritons, Tip-enhanced Raman spectroscopy

## Abstract

A novel tip-enhanced Raman spectroscopy setup with longitudinal field excitation generated by a plasmonic lens is investigated. A symmetry-breaking structure plasmonic lens that is expected to realize a strong longitudinal electric field focus has been designed to generate suitable excitation for enhancement in a tip antenna. The focusing performance of the plasmonic lens is theoretically simulated by the finite-difference time-domain method and experimentally verified by the detection of optical near-field distribution. A plasmonic lens assisted tip-enhanced Raman spectroscopy setup has been constructed and used to investigate specimens of carbon nanotubes. Tip-enhanced Raman spectra with distinct excitation wavelengths show similar Raman shifts but different intensities. Experimental results presented in this paper demonstrate that the Raman signal is considerably enhanced. It indicates that the novel tip-enhanced Raman spectroscopy configuration is feasible and is a promising technique for tip-enhanced Raman spectroscopy measurements and characterizations.

## Background

The optical antenna properties of metallic nanostructures attract considerable interest for a wide range of applications [1-4], in particular, due to such antennae featuring high confinement and strong enhancement of the local light-matter interaction [5,6]. For example, the strong local field enhancement on the metallic tip antenna is known to be responsible for tip-enhanced Raman spectroscopy (TERS) or related photochemical effects [7-10]. In the TERS technique, the signal enhancement near the antenna is due to the excitation of localized surface plasmons (LSPs) and the lightning-rod effect [11-13]. Both effects are highly sensitive to the polarization of the excitation optical field corresponding with the axis of the tip. The optical antenna theory reveals that, for a sharp metallic conical tip the longitudinal electrical field (LEF), (i.e., the optical field with its electric vector oriented along the z-axis, i.e., the axis of the tip), is significantly related to the magnitude of the field enhancement [13]. Firstly, LEF excitation satisfies the wave-vector matching condition for effective LSPs coupling. LEF excitation also concentrates the free electrons at the apex of the tip. Therefore, a strong LEF is required for efficient tip-enhancement excitation. In a conventional transmission-mode TERS setup, an objective lens is always utilized to focus the incident beam on the tip apex for excitation. With the view to obtaining a tightly focused LEF as the ideal excitation field, a higher order vector beam - such as radially polarized beam - is indispensable.

In this work, a metallic plasmonic lens (PL) with the symmetry-breaking structure is specifically designed for focusing the surface evanescent wave and generating the focused LEF which dominates the excitation field [14] under illumination of a linearly polarized beam. The focusing property of the longitudinal field by the PL is theoretically and experimentally investigated. It is proved and demonstrated that the PL is suitable for providing the LEF excitation for the local field enhancement on a tip antenna. Hence, the PL is utilized in the illumination configuration of the TERS setup instead of a conventional objective lens. It is used to realize focused LEF with linearly polarized incident beam. It makes the TERS setup more compact and easier to operate. That is to say, the PL-based TERS illumination frees the configuration from not only the modulation of the incident beam into higher-order polarization states but also the optical axis alignment of the illumination configuration with the beam. Additionally, it is of benefit to an ultra-compact configuration and the lab-on-a-chip technique. TERS experiments on carbon nanotube specimens are carried out. The results presented below indicate that the local Raman scattering signal from the specimen is significantly enhanced. It proves that the PL-assisted TERS setup is feasible and promising. Furthermore, the performances of the TERS setup under two distinct wavelengths of 632.8 and 532 nm are comparatively demonstrated and discussed. The results not only provide a deep understanding of the underlying physical mechanism of the PL-assisted TERS but also guide the optimization and development of the system for further applications.

## Methods

### Design of PL for TERS excitation

The vectorial feature is a significant physical property of the focusing optical field. In particular, the LEF has been attracting increasing attention during the past decades, due to its peculiarities and application prospects [15-17]. Especially, it is attractive in the optical antenna excitation, e.g., the tip-enhancement excitation of TERS. Specifically designed PLs are capable of concentrating the evanescent components of the incident light in the near-field and producing a LEF dominant focus with sub-diffraction-limit resolution.

**Figure 1:**
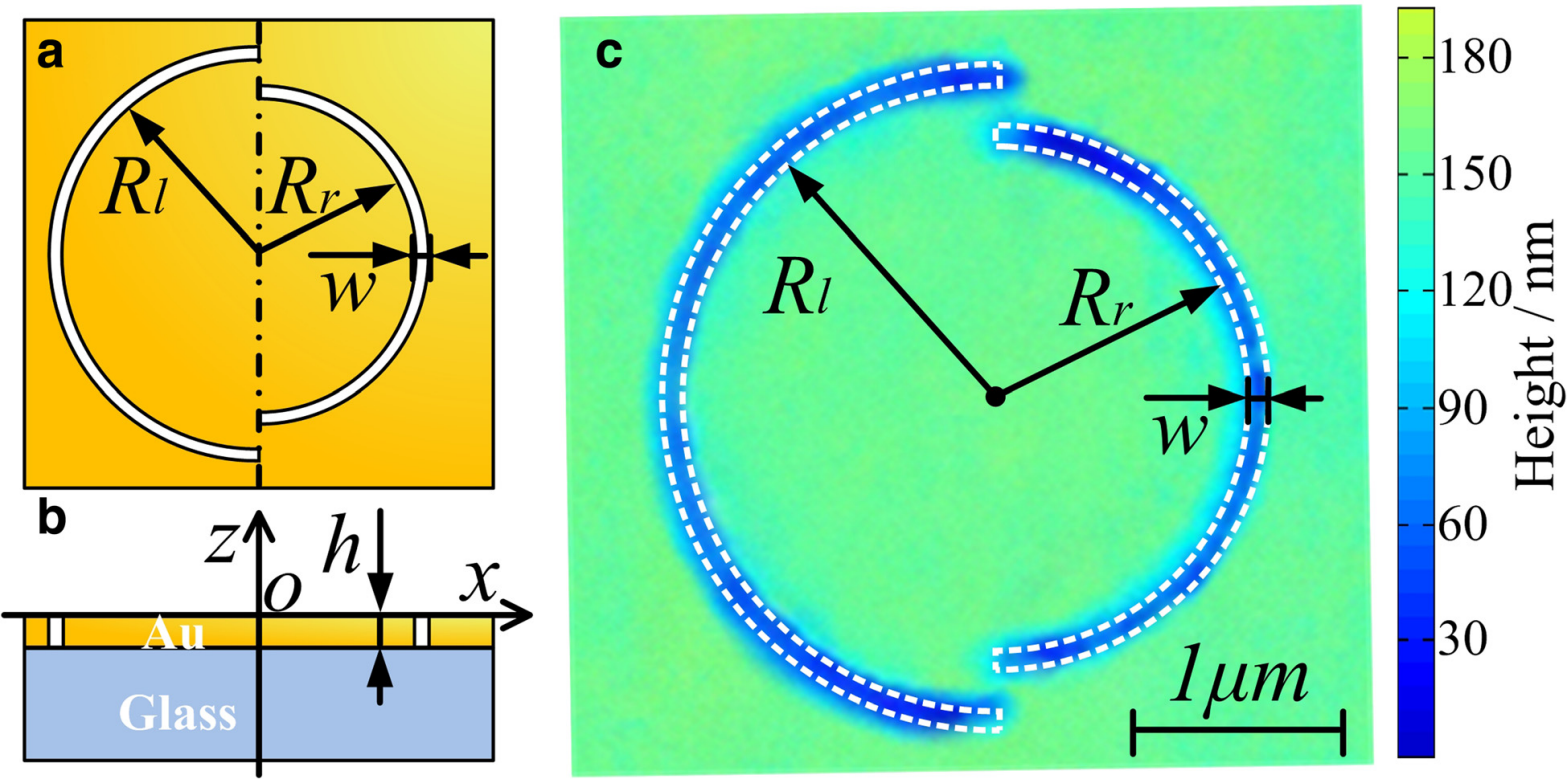
Schematics of PL structure consisting of a couple of semi-annular slits. In the *xy* plane **(a)** and in the *xz* plane **(b)**, *R*_*l*_ = 1.55 μm, *R*_*r*_ =1.25 μm, *w* = 100 nm, and *h* = 150 nm. Topographic image of the PL detected with an atomic force microscopy **(c)**.

Here, a PL with symmetry-breaking structure is specifically designed for the tightly confined LEF generation. The geometry of the PL is illustrated in Figure 1a,b. It consists of a couple of concentric semi-annular nano-slits, with a mismatched radius, corrugated on a gold film with 150 nm thickness. The width of slits, w, is 100 nm. The thickness of the gold film is a lot thicker than the skin depth for resisting the direct incident light, except in the area where the slits are fabricated. The sharp edges of the sub-wavelength slits act as plasmonic launchers to partially couple the portions of light with the electric field perpendicular to them into surface plasmon polaritons (SPPs) [18]. Each semi-annular edge can be regarded as a series of SPPs point sources. When the linearly polarized excitation beam is normally incident on a semi-annular slit, the excited SPP waves will propagate on the film surface and concentrate with interference in the center of the semi-annular [18-20]. While considering the concentric semi-annular slits on both left and right sides, the relationship of SPP waves’ phases launched from both sides should be taken into account regarding the interference. Under linearly polarized beam illumination, there is a π-phase difference in the initial phases of the LEF between the counter-propagating SPP waves generated by the two sides [18]. Thus, here, a radius mismatch is introduced to provide unequal SPP wave propagating lengths for compositing the initial phase difference. The inner radius of the semi-annular slit on the left side (*R*_*l*_) is 1.55 μm and on the right side (*R*_*r*_) is 1.25 μm. The radius mismatch between the two sides (*R*_*l*_ − *R*_*r*_) is approximately half a wavelength of the SPP wave propagating on the air-gold interface under 632.8-nm laser excitation. Thus, the initial phase difference between the two sides is compensated by the symmetry-breaking structure providing unequal SPP wave propagating lengths. That is to say, the radius mismatch regulates the total phase for constructive interference of LEF by breaking the axis symmetry of the structure about the axis perpendicular to the electric field of incident.

### Focusing property of the PL

**Figure 2:**
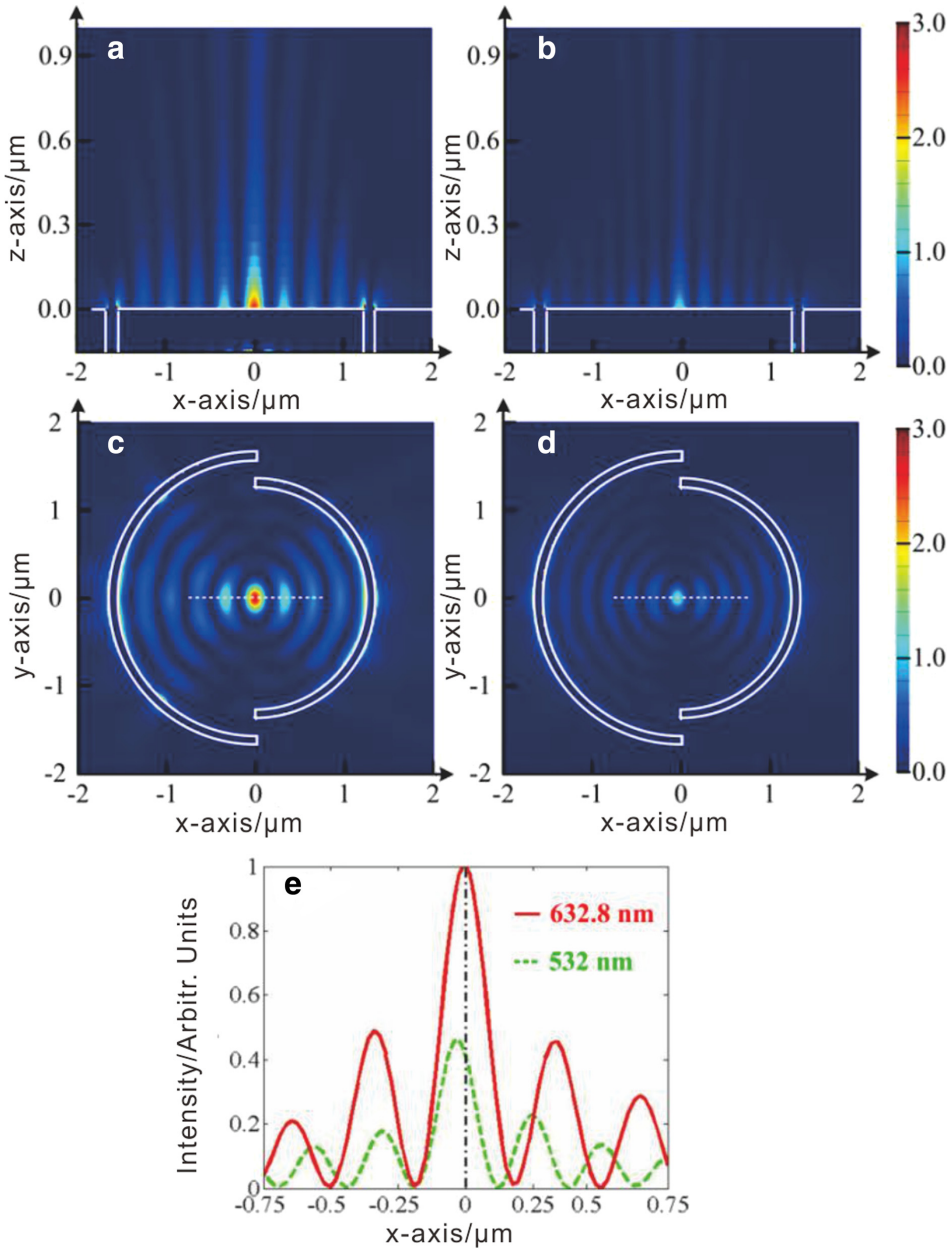
Calculated LEF intensity distributions of the PL under linearly polarized (x-axis) beam illumination. With two different wavelengths of 632.8 **(a, c)** and 532 nm **(b, d).** Panels **(a)** and **(b)** represent the LEF intensity distributions in the xz plane (*y* = 0), and panels **(c)** and **(d)** in the xy plane (*z* = 0). Intensity cross-section **(e)** along the *x*-axis in the central region of the PL (which is denoted by the white dashed lines in panels **(c)** and **(d)**.

In order to investigate the light-matter interaction, the finite-difference time-domain (FDTD) (FDTD Solution, Lumerical, Vancouver, Canada) method is employed to simulate the dynamics of the electromagnetic field. In FDTD modeling, the structure and parameters of the PL are presented as above. The PL is illuminated by a plane wave normally incident along the z-axis at the bottom. The incident wave is linearly polarized and the electric field is parallel to the x-axis. The simulations are performed at two different wavelengths of 632.8 and 532 nm, respectively. As showed in Figure 2, the LEF intensity distributions of the PL are calculated and presented. Figure 2a,c corresponds to the 632.8-nm illumination, while Figure 2b,d corresponds to the 532-nm illumination.

The focused LEF is formed to a dense bright spot and several weaker side lobes in the central region of the PL either with 632.8 or 532 nm illumination. With 632.8-nm illuminations, the maximum intensity of the focused LEF is about two times stronger than that with 532-nm illumination. For 632.8-nm illuminations, the full width at half maximum of the focusing spot is about 170 nm in the x-axis direction, beyond the diffraction limit. It is further demonstrated that the induced electric field is more confined in the x-direction than in the y-direction. Since the PL is designed for focusing 632.8-nm incident beams, under this illumination the focus spot is located at the center of the PL. While under the green laser illumination, there is about 30-nm deviation of the position of the spot along the x-axis. It is explained by the fact that that the period of SPP wave changed with distinct illumination wavelengths and consequently the phase may not be fully compensated at the center.

**Figure 3:**
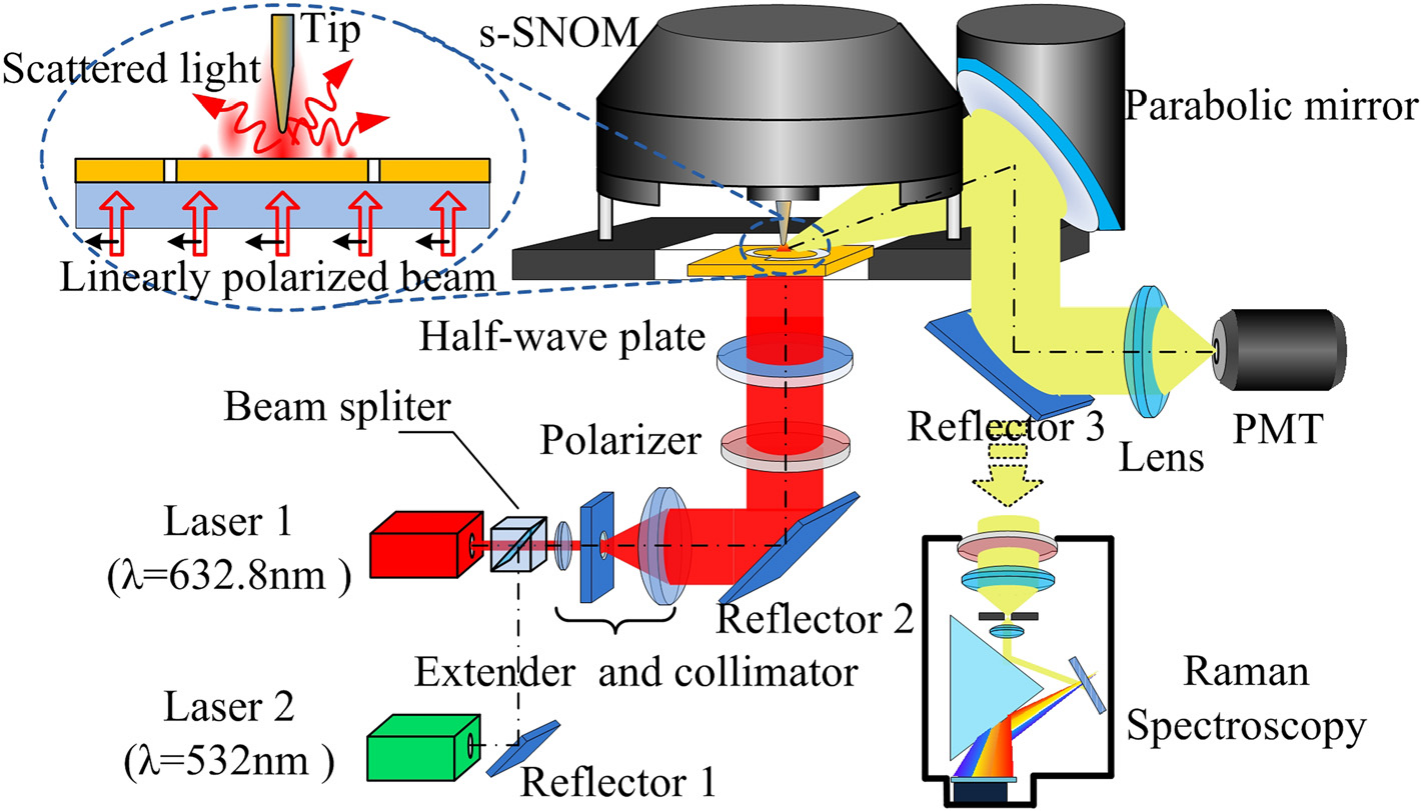
Experimental setup of the PL optical field detection and TRES with PL excitation.

According to the theoretical design, the PL was fabricated on a smooth gold film with 150 nm thickness evaporated onto a glass substrate. Patterns with a 100-nm-slit width were then milled through the film using a focused ion beam. The topography of the PL was imaged with an atomic force microscopy (AFM) (NT-MDT, NTEGRA, Moscow, Russia) and indicated in Figure 1c. Then, experiments were conducted to demonstrate the focusing performance of the PL. The LEF distributions were characterized using a scattering-type scanning near-field optical microscope (s-SNOM). The experimental setup is indicated schematically in Figure 3. A collimated linearly polarized laser beam, with along x-axis polarization, was incident from the bottom glass side on the PL structure. After interacting with the PL, the LEF intensity was detected with a silver-coated apertureless tip on the opposite side. The scattered light was gathered by a parabolic mirror and detected with a photomultiplier tube. By raster scanning, the tip over the central region of the PL surface, the near-field optical intensity distribution was obtained. In this experiment, an s-SNOM was utilized to detect the LEF intensity distribution. It is because apertureless scattering tips are more sensitive to the LEF. These tips ensure that the obtained signal is mainly from the LEF [21,22]. Two different incident beam wavelengths were respectively used. They were the 632.8-nm illumination, the operating wavelength of the PL as designed, and the 532-nm illumination, the comparison excitation wavelength. The LEF distributions were experimentally detected within 0.75 × 0.75 μm area in the center of the PL.

**Figure 4:**
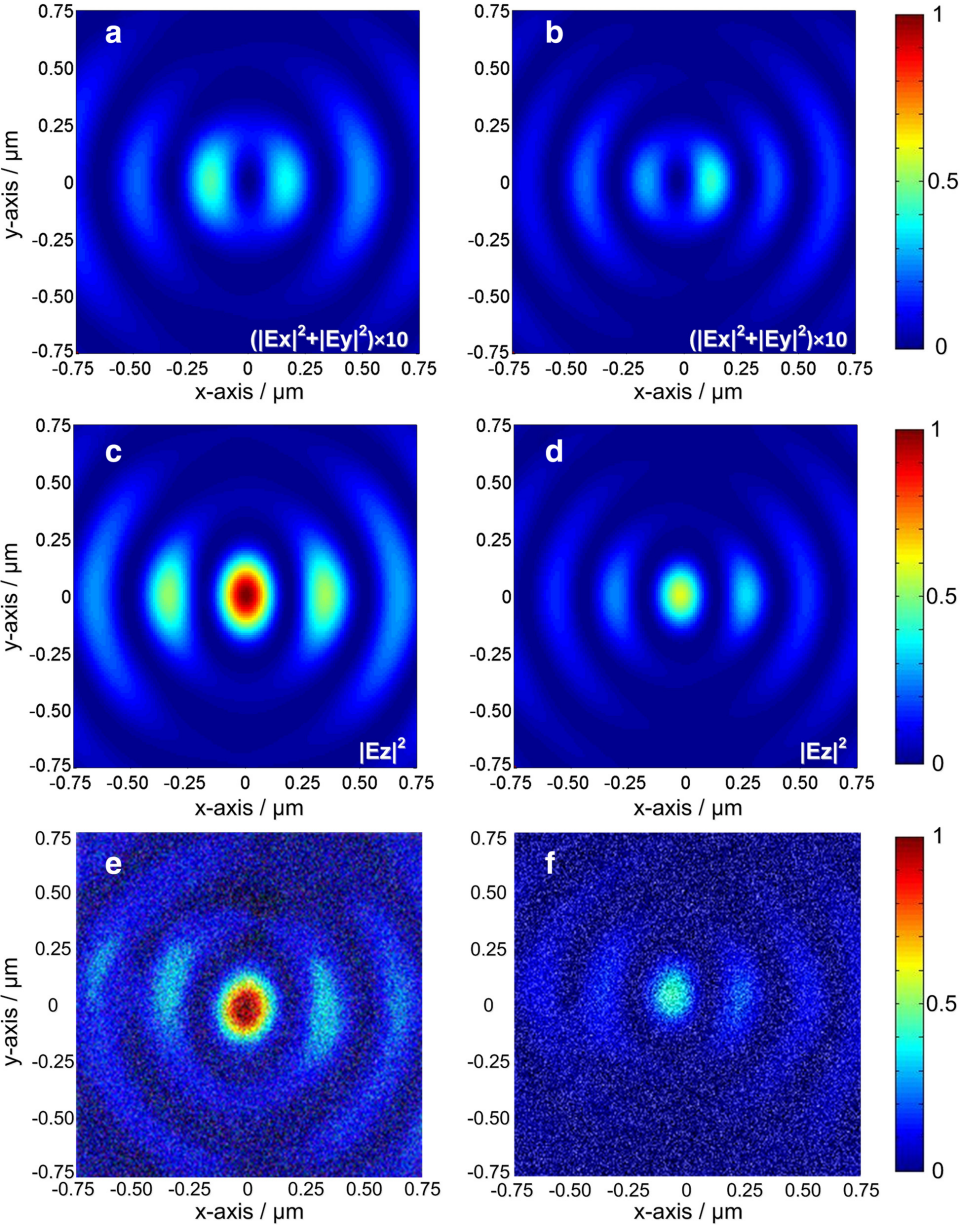
Calculated and experimentally measured optical field distribution from the PL. Calculated transverse electric field (|*Ex*|^2^ + |*Ey*|^2^) magnified ten times distribution **(a, b)** and LEF (|*Ez*|^2^) distribution **(c, d)**. Experimentally measured optical field distribution **(e, f)**. Panels **(a), (c),** and **(e)** correspond to 632.8-nm incident wavelength; panels **(b), (d),** and **(f)** correspond to 532 nm-incident wavelength.

The experimental results are presented in Figure 4e at 632.8-nm illumination and Figure 4f at 532-nm illumination. For comparison, calculated LEF distributions in the center of PL are shown in Figure 4c,d. In order to provide for a more precise optical near-field distribution, the actual PL structure was further used in the FDTD simulation. The AFM topographic image of the PL is imported to form the structural model. The simulation parameters are consistent with the experimental conditions.

The experimental result shown in Figure 4e,f agrees well with theoretical calculation shown in Figure 4c,d. It verifies the focusing performance of the PL. The optical field distribution shows an elliptic bright spot in the lens center and several arc-like side-lobes on both sides. As the normalized experimental results shown, the maximum intensity under 632.8-nm illuminations is comparatively strong than that under 532-nm illuminations.

### TERS experiments with PL-focused longitudinal field excitation

As its focusing performance was verified, the PL was introduced to a TERS illumination configuration instead of a conventional objective lens to construct a novel setup [23]. It served as a LEF-focusing component in the illumination configuration to concentrate incident energy and generate the excitation optical field. The TERS setup was built up based on the same platform of the s-SNOM shown in Figure 3. A single-walled carbon nanotubes (SWCNTs) sample was immobilized in the center of the PL surface. That is, the PL was utilized as a substrate at the same time. The tip was aligned above the center of the PL and regulated in the near-field of the sample surface. The excitation beam focused by the PL effectively interacted with the tip apex and strongly enhanced the local Raman signal of the sample. The tip-enhanced Raman signal was gathered by a parabolic mirror and then guided to a Raman spectroscope (Renishaw, InVia, Gloucestershire, UK) for spectral analysis.

## Results and discussion

**Figure 5:**
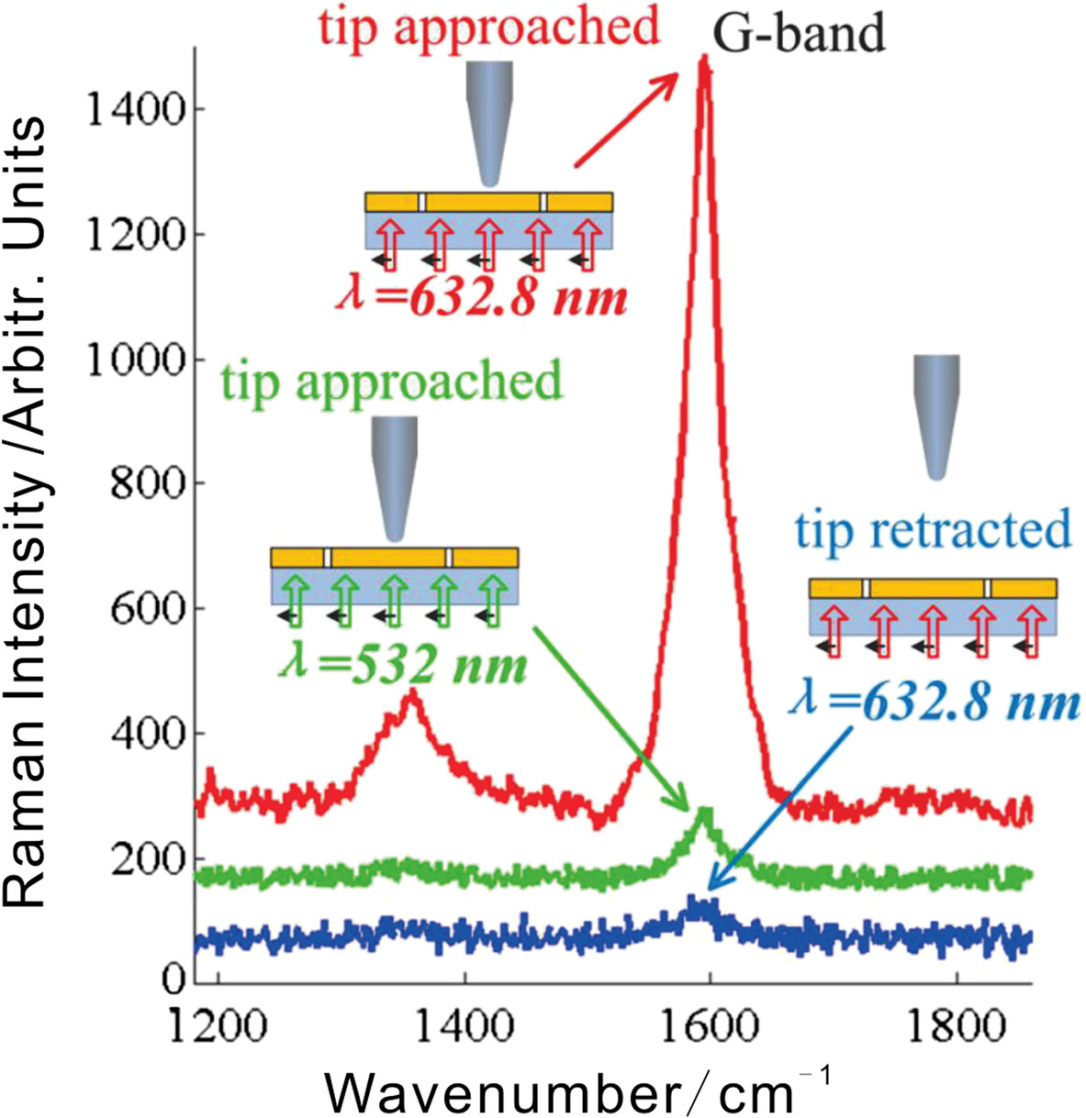
Tip-enhanced and far-field Raman spectra detected with the tip approached and retracted, respectively.

Using the PL-based setup, TERS experiments on SWCNTs sample were performed. Two lasers at distinct wavelengths of 632.8 and 532 nm were successively used as the excitation sources. The intensities of the different wavelength laser beam incident on the PL are adjusted to equal strength, 2 mW. With the tip approached, the tip-enhanced Raman spectra of the SWCNTs were respectively detected under each wavelength excitation. As shown in Figure 5, the red curve reflects the tip-enhanced Raman spectrum upon 632.8-nm excitation and the green curve corresponds to 532-nm excitation. The blue curve in Figure 5 shows the far-field Raman spectrum of the SWCNTs with the tip retracted under 632.8-nm excitation. For ease of viewing, the Raman spectra were offset. The detected Raman signal around the feature Raman shift of SWCNTs in G-band (approximately 1,590 cm^−1^) is selected to evaluate the performance of the TERS setup.

As shown in Figure 5, with the tip approached, the near-field Raman signals are strongly enhanced by the PL-tip system. The enhancement factor for the G-band intensity under 632.8-nm excitation is calculated to be about 3 × 10^3^, taking into consideration the different excitation areas. The enhancement, on the one hand, is resulting from the tip enhancement excited at the metallic tip apex by the focused LEF. And on the other hand, since the sample is sandwiched between two metallic interfaces of the tip and PL, much stronger local enhancement of the near-field Raman signal is achieved with gap-mode TERS [24]. Furthermore, the tip acts as an antenna enhancing both the incident and scattered optical field, so the local enhancement of the Raman intensity is proportional to the fourth power of the electric field [25]. Therefore, the effective enhancement of the PL-based TERS setup is ensured. Noticeably, the tip-enhanced Raman intensity with 632.8-nm excitation is about 15 times of that with 532-nm excitation. This is presumably related to the different LEF intensities and the excitation coefficients of the PL-tip system under distinct excitation wavelengths. Either the 632.8- or 532-nm laser that incident on the PL can be focused into a dense LEF dominant focus. That is to say, the tightly focused LEF can be realized under the two different wavelengths incident light with this same PL. However, the intensity of the focused central lobe is apparently much stronger with 632.8-nm illumination than that with 532-nm illumination. With 632.8-nm illuminations, the maximum intensity of the focused LEF is about two times stronger than that with 532-nm illumination. After introducing the tip, the electric field enhancement can be drastically further improved. In this research, a silver-coated AFM tip is used to perform the TERS experiments. Usually, the green light (mostly 532-nm laser) would be an appropriate choice as the excitation for the silver tips because the silver tips exhibit a LSP resonance in this wavelength region. However, in the case of the PL-tip coupling system, the TERS experiment results show that the tip-enhanced Raman intensity with 632.8-nm excitation is about 15 times of that with 532-nm excitation. In this case, we should consider the competing effect of the intensity of the excitation field focused by the PL and the LSP resonance-induced local field enhancement. With 632.8-nm illumination, the excitation field that reaches the tip apex is stronger than that with 532-nm illumination. And the TERS results reveal a significantly strengthened TERS enhancement under 632.8-nm laser excitation. The TERS enhancement is about 15 times of that with the 532-nm excitation. It can be assumed that here the lightning-rod effect plays an important role in the tip enhancement. With different excitation wavelengths of 632.8 and 532 nm, the tip-enhanced Raman spectra shows similar Raman shifts at about 1,590 cm^−1^. It confirmed that the Raman shift does not depend on the excitation wavelength.

## Conclusions

In conclusion, a novel TERS setup based on PL excitation was built up. The PL with symmetry-breaking structure was designed to focus linearly polarized incident beam and generate confined LEF. The focusing performance of the PL was experimentally demonstrated by detecting the LEF distribution with an s-SNOM. The experimental results were confirmed by FDTD simulations and analytical calculations. The designed PL has the advantage that it is able to generate a spatially concentrated illumination field with strong LEF component. It is rather suitable for extreme enhancement of TERS excitation. Therefore, the PL was introduced to the TERS illumination configuration as a focusing device for excitation. The PL-based TERS setup was utilized to investigate a SWCNTs sample with different excitation wavelengths. Experimental results indicate that the Raman scattering signal is significantly enhanced owing to tip enhancement and gap-mode enhancement. It proves experimentally that the combination of a PL-focused longitudinal excitation field with a metallic tip in a TERS system is a promising method. Additionally, the use of a PL in a TERS setup as a concentrating device as well as a substrate is also one step further towards realizing an ultra-compact configuration and the lab-on-a-chip technique. Tip-enhanced Raman spectra with distinct excitation wavelengths show similar Raman shifts but different intensities. The tip-enhanced Raman intensity with 632.8-nm excitation is much stronger than that of 532-nm excitation. It can be ascribed to the different LEF intensities and the excitation coefficients of the PL-tip system under distinct excitation wavelengths.

## Abbreviations

LEF: Longitudinal electrical field
LSP: Localized surface plasmon
PL: Plasmonic lens
SPP: Surface plasmon polariton
SWCNT: Single-walled carbon nanotube
TERS: Tip-enhanced Raman spectroscopy
